# Supplementary material for: Feedback control in planarian stem cell systems
Source: BMC Syst Biol. 2016 Feb 13;10:17. doi: 10.1186/s12918-016-0261-8 (PMC4752765; doi:10.1186/s12918-016-0261-8)
Supplement: Supplementary file 1 — The supplementary materials contains the codes for both models. Four codes are included in one PDF document (so need to be identified and separated if used): C script for the full model; R script for visualizing output of the full model; C script for the simplified model; and R script for visualizing output of the simplified model. Computations were done on a 2012 MacBook Pro using the gcc compiler with home directory ’marco’. (PDF 323 kb) [file 12918_2016_261_MOESM1_ESM.pdf]

```
//Mangel et a BMC Systems Biology codes

//There are 4 codes here: C script for the full model; R script for output of the full model; C script for the simplified model; and R script for the simplified model


//Computations were done on a 2012 MacBook Pro using the gcc compiler with home directory 'marco'


//************************************************************************************************************************
//************************************************************************************************************************

//****************************************C Script for Full Model ****************************************************

//************************************************************************************************************************
//************************************************************************************************************************




#include <stdio.h>
#include <math.h>
#include <stdlib.h>
#include <time.h>



FILE * fptr;




int main()
{
        printf("Full model from Mangel et al BMC Systems Biology\n");




int I_d, i_d,i_y,i,Imax;
double s[40],mu_0,denom,p1,p2,p3,p0,m_r,m_d,phi_bar,w[40],Y[60],sum_w1,sum_w2,delta,gamma;
double Nbar[60],Dbar[5][60],Dtot[60],eta_N, Size_bar[60],alpha[5][60];
double Y_e,epsilon[5],N_c,f_N_mult,f_N,N_mult,sig_N,mu_tot;
double N,D[5],Size,n,d[5],f_D,mu_mult,rho_bar[5][60],rho[5],mu_D[5],mu_rho[5];
double sig_D,sig_rho;


//number of types of differentiated cells
I_d=3;

Imax=200000;
eta_N=0.25;

for(i_d=1;i_d<=I_d;++i_d)
{
epsilon[i_d]=1.0;
```

```
}

f_N_mult=0.15;
N_mult=0.35;


//parameters
p1=0.0001;
p2=0.0005;
p3=0.00015;
phi_bar=p1/p3;
//note that this defines what i to i+1 means.
p0=1-p1-p2-p3;

delta=2./3.;

///////// Note
m_r=1.0;
m_d=4.0;
gamma=0.8*m_d;


s[1]=.75;s[2]=1;s[3]=1;
mu_0=.00015;



//now define the w[i]
printf("Now get the w[i]\n");
for(i_d=1;i_d<=I_d;++i_d)
{
w[i_d]=phi_bar*(p2+2*p3);
w[i_d]=w[i_d]/I_d;
w[i_d]=w[i_d]/(1-exp(-s[i_d]*mu_0));
//printf("%6d\t\%E\t\%E\n",i_d,w[i_d],s[i_d]*mu_0);
}



sum_w1=0;
sum_w2=0;
for(i_d=1;i_d<=3;++i_d)
{
sum_w2=sum_w2 + w[i_d]*(1-exp(-s[i_d]*mu_0));
sum_w1=sum_w1+ w[i_d];
}
//printf("%10.4f\t\%10.4f\t\%10.4f\t\%10.4f\n",s[1],w[1],w[2],w[3]);





//ready for steady state analysis
printf("Ready for steady state analysis.  i_d=2 corresponds to gut system\n");
for(i_y=1;i_y<=30;++i_y)
{
Y[i_y]=30.0*i_y;

denom=m_r*(1+sum_w1)+ m_d*(p1+phi_bar*(p2+p3))-gamma*sum_w2;
```

```
Nbar[i_y]=Y[i_y]*exp(delta*log(w[1]))/denom;
Nbar[i_y]=exp((1/(1-delta))*log(Nbar[i_y]));
Dtot[i_y]=0;
//now get the Dbars
for(i_d=1;i_d<=3;++i_d)
{
Dbar[i_d][i_y]=w[i_d]*Nbar[i_y];
Dtot[i_y]=Dtot[i_y]+Dbar[i_d][i_y];
alpha[i_d][i_y]=(1/Dbar[i_d][i_y])*((1/phi_bar)-1);
}
//Size_bar[i_y]=exp(-4.3839+.49713*log(Nbar[i_y]+Dtot[i_y]));
Size_bar[i_y]=exp(-3.8373+.46582*log(Dtot[i_y]));

for(i_d=1;i_d<=I_d;++i_d)
{
rho_bar[i_d][i_y]=Dbar[i_d][i_y]/Dtot[i_y];
}

printf("%E\t\%E\t\%E\t\%E\t\%E\t\%E\t\%E\n",Y[i_y],Size_bar[i_y],Nbar[i_y]/Dtot[i_y],Dbar[1]
[i_y]/Dtot[i_y],Dbar[2][i_y]/Dtot[i_y],Dbar[3][i_y]/Dtot[i_y],alpha[1][i_y]);
}


fptr=fopen("NDPQ1 Steady State.txt","w");
for(i_y=1;i_y<=30;++i_y)
{
fprintf(fptr,"%10.4f\t\%E\t\%E\t\%E\t\%E\t\%E\t\%E\t\%E\t\%E
\n",Y[i_y],Size_bar[i_y],Nbar[i_y]/Dtot[i_y],rho_bar[1][i_y],rho_bar[2][i_y],rho_bar[3][i_y],alpha[1]
[i_y],alpha[2][i_y],alpha[2][i_y]);
}
fclose(fptr);

//***************************************************************************

printf("Ready for growth and remoulding wo resource constraint\n");

int counter;
fptr=fopen("Grow No Constraints.txt","w");

i_y=15;
Y_e=Y[15];
N=0.2*Nbar[i_y];
D[1]=0.2*Dbar[1][i_y];
D[2]=0.2*Dbar[2][i_y];
D[3]=0.2*Dbar[3][i_y];
//Size=exp(-4.3839+.49713*log(N+D[1]+D[2]+D[3]));
Size=exp(-3.8373+.46582*log(D[1]+D[2]+D[3]));
printf("Initial conditions\n");
printf("%E\t\%E\t\%E\t\%E\t\%10.4f\n",N,D[1],D[2],D[3],Size);

counter=0;
N_c=f_N_mult*Nbar[i_y];
sig_N=f_N_mult*Nbar[i_y];


counter=3999;
for(i=0;i<=Imax;++i)
{
n=N;
```

```
d[1]=D[1];d[2]=D[2];d[3]=D[3];
for(i_d=1;i_d<=I_d;++i_d)
{
rho[i_d]=d[i_d]/(d[1]+d[2]+d[3]);
}


if(i==70000)
{
printf("Switch food\n");
i_y=10;
Y_e=Y[i_y];
N_c=f_N_mult*Nbar[i_y];
sig_N=f_N_mult*Nbar[i_y];
//this becomes very important for growth wo resource constraints
}


if(i==140000)
{
printf("Switch food\n");
i_y=16;
Y_e=Y[i_y];
N_c=f_N_mult*Nbar[i_y];
sig_N=f_N_mult*Nbar[i_y];
}



f_N=exp((n-N_c)/sig_N);
f_N=f_N/(1+f_N);

f_D=-1;
for(i_d=1;i_d<=I_d;++i_d)
{
if(f_D<1/(1+alpha[i_d][i_y]*d[i_d]))
{
f_D=1/(1+alpha[i_d][i_y]*d[i_d]);
}
}

N=n*(1+p1-p3*f_D*f_N);

denom=0;
for(i_d=1;i_d<=I_d;++i_d)
{
denom=denom + (Dbar[i_d][i_y]/d[i_d]);
}

mu_tot=0;
for(i_d=1;i_d<=I_d;++i_d)
{

sig_D=0.15*Dbar[i_d][i_y];
sig_rho=0.15*rho_bar[i_d][i_y];

//sigmoida multiplier
mu_D[i_d]=exp( (d[i_d]-Dbar[i_d][i_y])/sig_D);
mu_D[i_d]=mu_D[i_d]/(1+mu_D[i_d]);
```

```
mu_rho[i_d]=exp( (rho[i_d]-rho_bar[i_d][i_y])/sig_rho);
mu_rho[i_d]=mu_rho[i_d]/(1+mu_rho[i_d]);

mu_mult=0 + (mu_D[i_d] + mu_rho[i_d])*s[i_d]*mu_0;

//algebraic multiplier
//mu_mult= d[i_d]/Dbar[i_d][i_y];
//mu_mult=exp(epsilon[i_d]*log(d[i_d]/Dbar[i_d][i_y]));
//mu_mult=mu_mult+exp(epsilon[i_d]*log(rho[i_d]/rho_bar[i_d][i_y]));
//mu_mult=0.5*mu_mult;


D[i_d]=d[i_d]*exp(-mu_mult);
D[i_d]=D[i_d]+n*(p2*f_D+2*p3*f_D*f_N)*( (Dbar[i_d][i_y]/d[i_d])/denom);
mu_tot=mu_tot + d[i_d]*(1-exp(-mu_mult));
}

mu_tot=mu_tot/(d[1]+d[2]+d[3]);
//Size=exp(-4.3839+.49713*log(N+D[1]+D[2]+D[3]));
Size=exp(-3.8373+.46582*log(D[1]+D[2]+D[3]));
counter=counter+1;
if(counter==4000)
{
printf("%6d\t\%10.4f\t\%E\t\%E\n",i,Size,mu_tot,n/(n+d[1]+d[2]+d[3]));
counter=0;
}
//printf("Printing to the file\n");
fprintf(fptr,"%6d\t\%10.4f\t\%E\t\%E\n",i,Size,mu_tot,n/(n+d[1]+d[2]+d[3]));
//end of time loop
}
fclose(fptr);

//****************************  Remoulding  ************************************


printf("Ready for remoulding after division wo resource constraint\n");


fptr=fopen("Remoulding No Constraints.txt","w");

i_y=15;
Y_e=Y[15];
N=0.5*Nbar[i_y];
D[1]=0.1*Dbar[1][i_y];
D[2]=0.3*Dbar[2][i_y];
D[3]=0.05*Dbar[3][i_y];
//Size=exp(-4.3839+.49713*log(N+D[1]+D[2]+D[3]));
Size=exp(-3.8373+.46582*log(D[1]+D[2]+D[3]));
printf("Initial conditions\n");
printf("%E\t\%E\t\%E\t\%E\t\%10.4f\n",N,D[1],D[2],D[3],Size);

counter=0;
N_c=f_N_mult*Nbar[i_y];
sig_N=f_N_mult*Nbar[i_y];


counter=3999;
for(i=0;i<=Imax;++i)
```

```
{
n=N;
d[1]=D[1];d[2]=D[2];d[3]=D[3];
for(i_d=1;i_d<=I_d;++i_d)
{
rho[i_d]=d[i_d]/(d[1]+d[2]+d[3]);
}



f_N=exp((n-N_c)/sig_N);
f_N=f_N/(1+f_N);

f_D=-1;
for(i_d=1;i_d<=I_d;++i_d)
{
if(f_D<1/(1+alpha[i_d][i_y]*d[i_d]))
{
f_D=1/(1+alpha[i_d][i_y]*d[i_d]);
}
}

N=n*(1+p1-p3*f_D*f_N);

denom=0;
for(i_d=1;i_d<=I_d;++i_d)
{
denom=denom + (Dbar[i_d][i_y]/d[i_d]);
}

mu_tot=0;
for(i_d=1;i_d<=I_d;++i_d)
{
//mu_mult=exp(epsilon[i_d]*log(d[i_d]/Dbar[i_d][i_y]));
//mu_mult=mu_mult+exp(epsilon[i_d]*log(rho[i_d]/rho_bar[i_d][i_y]));
//mu_mult=0.5*mu_mult;
//mu_D[i_d]=exp(epsilon[i_d]*log(d[i_d]/Dbar[i_d][i_y]));
//mu_rho[i_d]=exp(epsilon[i_d]*log(rho[i_d]/rho_bar[i_d][i_y]));

sig_D=0.05*Dbar[i_d][i_y];
sig_rho=0.05*rho_bar[i_d][i_y];

//sigmodical multipler
mu_D[i_d]=exp( (d[i_d]-Dbar[i_d][i_y])/sig_D);
mu_D[i_d]=mu_D[i_d]/(1+mu_D[i_d]);

mu_rho[i_d]=exp( (rho[i_d]-rho_bar[i_d][i_y])/sig_rho);
mu_rho[i_d]=mu_rho[i_d]/(1+mu_rho[i_d]);

mu_mult=0 + (mu_D[i_d] + mu_rho[i_d])*s[i_d]*mu_0;

//algebraic multiplier
//mu_mult= d[i_d]/Dbar[i_d][i_y];
D[i_d]=d[i_d]*exp(-mu_mult);
D[i_d]=D[i_d]+n*(p2*f_D+2*p3*f_D*f_N)*( (Dbar[i_d][i_y]/d[i_d])/denom);
mu_tot=mu_tot+d[i_d]*(1-exp(-mu_mult));
}
mu_tot=mu_tot/(d[1]+d[2]+d[3]);
```

```
//Size=exp(-4.3839+.49713*log(N+D[1]+D[2]+D[3]));
Size=exp(-3.8373+.46582*log(D[1]+D[2]+D[3]));
counter=counter+1;
if(counter==4000)
{
printf("%6d\t\%10.4f\t\%E\t\%E\n",i,Size,mu_tot,n/(n+d[1]+d[2]+d[3]));
counter=0;
}
//printf("Printing to the file\n");
fprintf(fptr,"%6d\t\%10.4f\t\%E\t\%E\t\%E\t\%E\t\%E\t\%E\t\%E\n",i,Size,mu_tot,n/(n
+d[1]+d[2]+d[3]),rho[1],rho[2],rho[3],mu_D[1]+mu_D[2]+mu_D[3],mu_rho[1]+mu_rho[2]+mu_rho[3]
);
//end of time loop
}
fclose(fptr);
        return 0;
}




//****************************************************************************************************************
//****************************************************************************************************************

//************************************ End of C Script for Full Model
****************************************************

//****************************************************************************************************************
//****************************************************************************************************************







//****************************************************************************************************************
//****************************************************************************************************************

//************************************R Script for Full Model ************************************************

//****************************************************************************************************************
//****************************************************************************************************************





A <-read.table("~marco/NDPQ1 Steady State.txt", header=FALSE)
B <-read.table("~marco/Grow No Constraints.txt", header=FALSE)
C <-read.table("~marco/Remoulding No Constraints.txt", header=FALSE)



#-- steady state size
quartz()
Y=A[,1]
Size=A[,2]

```

```
plot(Y,Size,type="l",xlab="Food level",ylab="Steady state size (mm)",lwd=3);

quartz()
alpha_1=A[,7]
alpha_2=A[,8]
alpha_3=A[,9]
plot(Y,log(alpha_1),type="l",xlab="Food level",ylab="Strength of Feedback (log scale)",lwd=3);
lines(Y,log(alpha_2)+0.1,type="l",xlab="Food level",ylab="alpha",lty=4,col="blue");
lines(Y,log(alpha_3),type="l",xlab="Food level",ylab="alpha",lty=6,lwd=3,col="red");


quartz()
#-- growing under no constraints
tyme=B[,1]
Size=B[,2]
mu_T=B[,3]
frac_N=B[,4]

par(mfrow=c(3,1))

plot(tyme/10000,Size,type="l",xlab="Scaled time",ylab="Size (mm)",lwd=3);

plot(tyme/10000,mu_T,type="l",xlab="Scaled time",ylab="Total mortality",lwd=3);

plot(tyme/10000,frac_N,type="l",xlab="Scaled time",ylab="Fraction neoblasts",lwd=3);


quartz()
#-- remoulding under no constraints
tyme=C[,1]
Size=C[,2]
mu_T=C[,3]
frac_N=C[,4]
rho1=C[,5]
rho2=C[,6]
rho3=C[,7]
mu_D=C[,8]
mu_rho=C[,9]

par(mfrow=c(4,1))

plot(tyme/10000,Size,type="l",xlab="Scaled time",ylab="Size (mm)",lwd=3,ylim=c(12,35));


#plot(tyme,mu_rho,type="l",col="green",xlim=c(0,200000),ylim=c(0,2));
#lines(tyme,mu_D,type="l");

plot(tyme/10000,mu_T,type="l",xlab="Scaled time",ylab="Total mortality",lwd=3)

plot(tyme/10000,frac_N,type="l",xlab="Scaled time",ylab="Fraction neoblasts",lwd=3,ylim=c(0.1,0.5))


plot(tyme,rho2,type="l",col="red",xlim=c(0,10000),xlab="Time",ylab="Fraction Differentiated Cells",lwd=3,ylim=c(0.0,0.8))
lines(tyme,rho1,type="l",lwd=3);
lines(tyme,rho3,type="l",col="blue",lwd=3)
```

```
//*******************************************************************************************************************
//*******************************************************************************************************************

//************************************ End of R Script for Full Model **************************************************

//*******************************************************************************************************************
//*******************************************************************************************************************





//*******************************************************************************************************************
//*******************************************************************************************************************

//************************************C Script for the Simplified Model************************************

//*******************************************************************************************************************
//*******************************************************************************************************************




#include <stdio.h>
#include <math.h>
#include <stdlib.h>
#include <time.h>



FILE * fptr;




int main()
{
	printf("Simplifed model from Mangel et al BMC Systems Biology\n");

double a_1,a_2,a_3, q_1,q_2,q_3,q_4, q_5,q_12,q_23,q_34, q_45,q, mu_1,qp,q_mu;
double p1,p2,p3,p0,kq,gamma,mu_0,eta,Omega;
double Y[50],Dbar[50],Nbar[50],Qmax[50],Qbar[50],m_r,m_d,alpha[50],f_0,Size_bar[50],f_N,f_N_mult,f_g, sig_N,N_mult, delta;
int i_y,i_q,Imax,i,I_q,counter,i_n,i_d;

Imax=200000;
I_q=50;

double N[Imax+1],D[Imax+1],Q[Imax+1],N_c,Y_e,n,d,Size[Imax+1],mu_1max,Q_max,f_D_ex, f_N_ex;




//********************  Ready for Steady State Results *******************
printf("Ready for steady state results\n");
```

```
//parameters
p1=0.0001;
p2=0.0005;
p3=0.00015;
//note that this defines what i to i+1 means.
p0=1-p1-p2-p3;
kq=20;

///////// Note
m_r=1.0;
m_d=4.0;


//multipler for the bandwidth on f_N
f_N_mult=0.15;
N_mult=0.35;

//fraction of D's that are gut
f_g=.95;

//Dont do this here. Some more thinking needed!

//food gathering exponent
delta = 2.0/3.0;

//other parameters
gamma=0.8*m_d;
Omega=p1*(p2+p3)/(p3);
Omega=Omega+p1;
//now fix mu to give 25% neoblasts
mu_0=-log(1 -  (p1*(p2+2*p3)/(3*p3)));
mu_1max=20*mu_0;
eta = (1.0-exp(-mu_0))*p3/(p1*(p2+2*p3));
printf("Mu giving 25 percent neoblasts at steady state is ");printf("%E\n",mu_0);


printf("Steady states\n");
for(i_y=1;i_y<=20;++i_y)
{
Y[i_y]=20*i_y;
Dbar[i_y]=Y[i_y]/(m_r*(1+eta)+m_d*eta*Omega-gamma*(1-exp(-mu_0)));
//Dbar[i_y]=Dbar[i_y]*Dbar[i_y]*Dbar[i_y];
Dbar[i_y]=exp( (1.0/(1.0-delta))*log(Dbar[i_y]));
Nbar[i_y]=eta*Dbar[i_y];
Qbar[i_y]=(Nbar[i_y]+Dbar[i_y])*m_d;
Qmax[i_y]=Qbar[i_y];
alpha[i_y]=(1/Dbar[i_y])*((p3/p1)-1);
f_0=1.0/(1+alpha[i_y]*Dbar[i_y]);
//Note: this is called f_N in later versions and in NDPQ1

Size_bar[i_y]=exp(-4.3839+.49713*log(Nbar[i_y]+Dbar[i_y]));

double lhs,rhs;
//printf("Check on steady states. Is N=eta*D\n");
//printf("%10.4f\n",N[0]-eta*D[0]);
//printf("Check on steady states, formulation of f_0");
//printf("%10.4f\n",f_0-(p1/p3));
//printf("Check on steady states. N equation\n");
```

```
//check on Dbar
lhs=exp(.333333333333*log(Dbar[i_y]));
rhs=Y[i_y]/( m_d*eta*Omega+m_r*(1+eta)-gamma*(1-exp(-mu_0)));


//check on f_0;
//lhs=1.0/(1+alpha[i_y]*Dbar[i_y]);
//rhs=p1/p3;


//check on Nbar
//lhs=Nbar[i_y];
//rhs=Nbar[i_y]*(1+p1-f_0*p3);



printf("%10.4f\t\%10.4f\t\%10.4f\t\%10.4f\t\%10.4f\t\%10.4f\n",Y[i_y],Nbar[i_y],Dbar[i_y],Qbar[i_y],Nbar[i_y]/(Nbar[i_y]+Dbar[i_y]),Size_bar[i_y]);
}



fptr=fopen("NDQ4 Steady State.txt","w");
for(i_y=1;i_y<=20;++i_y)
{
//Size-cell allometry: Size = exp(-4.3839+.49713 log(cells))

//printf("%6d\t\%10.4f\t\%10.4f\t\%10.4f\t\%10.4f\t\%10.4f\t\%10.4f\t\%10.4f\n",i_y,Y[i_y],Nbar[i_y],Dbar[i_y],Size_bar[i_y],Qbar[i_y],Nbar[i_y]/(Nbar[i_y]+Dbar[i_y]), f_0);
fprintf(fptr,"%10.4f\t\%10.4f\t\%10.4f\t\%10.4f\n",Y[i_y],Size_bar[i_y],Nbar[i_y]+Dbar[i_y],f_0);
}
fclose(fptr);

//******************************************  Ready for Growth and Regrowth ***************************

printf("Ready for growth and degrowth\n");
//pick a value of Y for food in the environment
i_y=12;
Y_e=Y[i_y];
printf("Starting food is ");printf("%10.4f\n",Y_e);



printf("i       Size[i]           q                   q_max           a_1           a_2         a_3          mu_1       f_N                   f_0           D[i]           N[i]/(N[i]+D[i])\n");
counter=0;
//initial conditions
N[0]=.2*Nbar[i_y];
D[0]=.2*Dbar[i_y];
Q[0]=.2*Qbar[i_y];
Size[0]=exp(-4.3839+.49713*log(N[0]+D[0]));

N_c=f_N_mult*Nbar[i_y];
```

```
sig_N=f_N_mult*Nbar[i_y];

fptr=fopen("Grow.txt","w");
counter=3999;
for(i=0;i<=Imax;++i)
{

if(i==20000)
{
printf("Switch food\n");
i_y=19;
Y_e=Y[i_y];
N_c=f_N_mult*Nbar[i_y];
sig_N=f_N_mult*Nbar[i_y];
}

if(i==70000)
{
printf("Switch food\n");
i_y=16;
Y_e=Y[i_y];
N_c=f_N_mult*Nbar[i_y];
sig_N=f_N_mult*Nbar[i_y];
}

if(i==150000)
{
printf("Switch food\n");
i_y=13;
Y_e=Y[i_y];
N_c=f_N_mult*Nbar[i_y];
sig_N=f_N_mult*Nbar[i_y];
}


if(i==-180000)
{
printf("Switch food\n");
i_y=19;
Y_e=Y[i_y];
N_c=f_N_mult*Nbar[i_y];
sig_N=f_N_mult*Nbar[i_y];
}
//now compute the multipliers on activities
n=N[i];d=D[i];q=Q[i];




f_N=exp((N[i]-N_c)/sig_N);
f_N=f_N/(1+f_N);


f_0=1/(1+alpha[i_y]*D[i]);

q_1=m_r*(n+d);
q_2=q_1+m_d*p1*n;
q_3=q_2+p2*f_0*n*m_d;
q_4=q_3+p3*f_0*f_N*n*m_d;
```

```
q_5=1.3*q_4;
q_mu=1.5*q_4;

q_12=0.5*(q_1+q_2);
q_23=0.5*(q_2+q_3);
q_34=0.5*(q_3+q_4);
q_45=0.5*(q_4+q_5);



	if(q<q_1)
{
	a_1=0;
}
if(q>=q_1)
{
	qp=q-q_1;
a_1=qp*qp/(qp*qp + (q_12-q_1)*(q_12-q_1));
}


if(q<q_2)
{
	a_2=0;
}

if(q>=q_2)
{
	qp=q-q_2;
a_2=qp*qp/(qp*qp + (q_23-q_2)*(q_23-q_2));
}

if(q<q_3)
{
	a_3=0;
}
if(q>q_3)
{
	qp=q-q_3;

a_3=qp*qp/(qp*qp + (q_34-q_3)*(q_34-q_3));
}

if(q>10*q_mu)
{
	mu_1=0;
}


if(q<q_mu)
{
	mu_1=1;
}
if(q>=q_mu)
{
if(q<=10*q_1)
{

	mu_1=(q_1-q_mu)*(q_1-q_mu)/(  (q_1-q_mu)*(q_1-q_mu)+ (q-q_mu)*(q-q_mu) );
```

```
//mu_1=1*exp(-q/q_mu);
}
}



mu_1=mu_1max*mu_1;



//a_1=1;
//a_2=1;
//a_3=1;
//f_0=p1/p3;
//f_N=1;


N[i+1]=N[i]*(1+p1*a_1-p3*f_0*f_N*a_3);

D[i+1]=D[i]*exp(-mu_0-mu_1)+f_0*(p2*a_2+2*p3*a_3*f_N)*N[i];

Q[i+1]=Q[i] + Y_e*exp(delta*log(D[i]))+gamma*D[i]*(1-exp(-mu_0-mu_1))-m_r*(N[i]+D[i]);
Q[i+1]=Q[i+1]-m_d*N[i]*(p1*a_1 + f_0*(p2*a_2+p3*a_3*f_N));

if(Q[i+1]<0){
        Q[i+1]=0;
        }

Q_max=m_d*(N[i+1]+D[i+1]);
if(Q[i+1]>Q_max){
        Q[i+1]=Q_max;
        }
Size[i+1]=exp(-4.3839+.49713*log(N[i+1]+D[i+1]));

counter=counter+1;
if(counter==4000)
{
printf("%6d\t\%10.4f\t\%E\t\%E\t\%E\t\%1E\t\%E\t\%E\t\%E\t\%10.4f\t\%E\t\%10.4f\n",i,Size[i],q,Q_max,a_1*p1,a_2*f_0*p2,a_3*f_0*f_N*p3,mu_1,f_N,f_0,D[i],N[i]/(N[i]+D[i]));
counter=0;
}

fprintf(fptr,"%6d\t\%10.4f\t\%10.4f\t\%10.4f\t\%10.4f\t\%10.4f\t\%10.4f\t\%10.4f\t\%10.4f\t\%10.4f\n",i,Size[i],a_1*p1,a_2*f_0*p2,a_3*f_0*f_N*p3,mu_1,N[i]/(N[i]+D[i]),Y_e,f_0,f_N);

//end of i loop
}
fclose(fptr);

//*******************************************************************************************************************************


//******************************** Now the excision experiment ***************************************

printf("Ready for excision\n");
//pick a value of Y for food in the environment
i_y=15;
```

```
Y_e=Y[i_y];
//printf("Starting food is ");printf("%10.4f\n",Y_e);



printf("i     Size[i]     q          q_max      a_1      a_2      a_3      mu_1      f_N          f_0      D[i]      N[i]/(N[i]+D[i])\n");
counter=0;
//initial conditions
N[0]=.3*Nbar[i_y];
D[0]=.3*Dbar[i_y];
Q[0]=.3*Qbar[i_y];
Size[0]=exp(-4.3839+.49713*log(N[0]+D[0]));

N_c=f_N_mult*Nbar[i_y];
sig_N=f_N_mult*Nbar[i_y];

fptr=fopen("Excision.txt","w");
counter=3999;
for(i=0;i<=Imax;++i)
{

if(i==70000)
{
printf("Excision\n");
D[i]=0.5*D[i];
}

//now compute the multipliers on activities
n=N[i];d=D[i];q=Q[i];



f_N=exp((N[i]-N_c)/sig_N);
f_N=f_N/(1+f_N);



f_0=1/(1+alpha[i_y]*D[i]);

q_1=m_r*(n+d);
q_2=q_1+m_d*p1*n;
q_3=q_2+p2*f_0*n*m_d;
q_4=q_3+p3*f_0*f_N*n*m_d;
q_5=1.3*q_4;
q_mu=1.5*q_4;

q_12=0.5*(q_1+q_2);
q_23=0.5*(q_2+q_3);
q_34=0.5*(q_3+q_4);
q_45=0.5*(q_4+q_5);


	if(q<q_1)
{
	a_1=0;
}
if(q>=q_1)
```

```
{
        qp=q-q_1;
a_1=qp*qp/(qp*qp + (q_12-q_1)*(q_12-q_1));
}


if(q<q_2)
{
        a_2=0;
}

if(q>=q_2)
{
        qp=q-q_2;
a_2=qp*qp/(qp*qp + (q_23-q_2)*(q_23-q_2));
}

if(q<q_3)
{
        a_3=0;
}
if(q>q_3)
{
        qp=q-q_3;

a_3=qp*qp/(qp*qp + (q_34-q_3)*(q_34-q_3));
}

if(q>10*q_mu)
{
        mu_1=0;
}


if(q<q_mu)
{
        mu_1=1;
}
if(q>=q_mu)
{
if(q<=10*q_1)
{

        mu_1=(q_1-q_mu)*(q_1-q_mu)/(  (q_1-q_mu)*(q_1-q_mu)+ (q-q_mu)*(q-q_mu) );
//mu_1=1*exp(-q/q_mu);
}
}



mu_1=mu_1max*mu_1;



//a_1=1;
//a_2=1;
//a_3=1;
//f_0=p1/p3;
//f_N=1;
```

```
N[i+1]=N[i]*(1+p1*a_1-p3*f_0*f_N*a_3);

D[i+1]=D[i]*exp(-mu_0-mu_1)+f_0*(p2*a_2+2*p3*a_3*f_N)*N[i];

Q[i+1]=Q[i] + Y_e*exp(delta*log(D[i]))+gamma*D[i]*(1-exp(-mu_0-mu_1))-m_r*(N[i]+D[i]);
Q[i+1]=Q[i+1]-m_d*N[i]*(p1*a_1 + f_0*(p2*a_2+p3*a_3*f_N));

if(Q[i+1]<0){
	Q[i+1]=0;
	}

Q_max=m_d*(N[i+1]+D[i+1]);
if(Q[i+1]>Q_max){
	Q[i+1]=Q_max;
	}
Size[i+1]=exp(-4.3839+.49713*log(N[i+1]+D[i+1]));

counter=counter+1;
if(counter==4000)
{
printf("%6d\t\%10.4f\t\%E\t\%E\t\%E\t\%1E\t\%E\t\%E\t\%E\t\%10.4f\t\%E\t\%10.4f\n",i,Size[i],q,Q_max,a_1*p1,a_2*f_0*p2,a_3*f_0*f_N*p3,mu_1,f_N,f_0,D[i],N[i]/(N[i]+D[i]));
counter=0;
}

fprintf(fptr,"%6d\t\%10.4f\t\%10.4f\t\%10.4f\t\%10.4f\t\%10.4f\t\%10.4f\t\%10.4f\%10.4f\t\%10.4f\n",i,Size[i],a_1*p1,a_2*f_0*p2,a_3*f_0*f_N*p3,mu_1,N[i]/(N[i]+D[i]),Y_e,f_0,f_N);

//end of i loop
}
fclose(fptr);

//*******************************************************************************************************************************

//******************************** Now the X-ray experiment **************************************

printf("Ready for X-ray\n");
//pick a value of Y for food in the environment
i_y=15;
Y_e=Y[i_y];
//printf("Starting food is ");printf("%10.4f\n",Y_e);




printf("i	Size[i]		q		q_max		a_1		a_2		a_3		mu_1		f_N		f_0		D[i]		N[i]/(N[i]+D[i])\n");
counter=0;
//initial conditions
N[0]=.25*Nbar[i_y];
D[0]=.25*Dbar[i_y];
Q[0]=.25*Qbar[i_y];
Size[0]=exp(-4.3839+.49713*log(N[0]+D[0]));

N_c=f_N_mult*Nbar[i_y];
```

```
sig_N=f_N_mult*Nbar[i_y];

fptr=fopen("Xray.txt","w");
counter=3999;
for(i=0;i<=Imax;++i)
{

if(i==70000)
{
printf("Xray\n");
N[i]=0.75*N[i];
}

//now compute the multipliers on activities
n=N[i];d=D[i];q=Q[i];


f_N=exp((N[i]-N_c)/sig_N);
f_N=f_N/(1+f_N);


f_0=1/(1+alpha[i_y]*D[i]);

q_1=m_r*(n+d);
q_2=q_1+m_d*p1*n;
q_3=q_2+p2*f_0*n*m_d;
q_4=q_3+p3*f_0*f_N*n*m_d;
q_5=1.3*q_4;
q_mu=1.5*q_4;

q_12=0.5*(q_1+q_2);
q_23=0.5*(q_2+q_3);
q_34=0.5*(q_3+q_4);
q_45=0.5*(q_4+q_5);


        if(q<q_1)
{
        a_1=0;
}
if(q>=q_1)
{
        qp=q-q_1;
a_1=qp*qp/(qp*qp + (q_12-q_1)*(q_12-q_1));
}


if(q<q_2)
{
        a_2=0;
}

if(q>=q_2)
{
        qp=q-q_2;
a_2=qp*qp/(qp*qp + (q_23-q_2)*(q_23-q_2));
}

if(q<q_3)
```

```
{
	a_3=0;
}
if(q>q_3)
{
	qp=q-q_3;

a_3=qp*qp/(qp*qp + (q_34-q_3)*(q_34-q_3));
}

if(q>10*q_mu)
{
	mu_1=0;
}


if(q<q_mu)
{
	mu_1=1;
}
if(q>=q_mu)
{
if(q<=10*q_1)
{

	mu_1=(q_1-q_mu)*(q_1-q_mu)/(  (q_1-q_mu)*(q_1-q_mu)+ (q-q_mu)*(q-q_mu) );
//mu_1=1*exp(-q/q_mu);
}
}



mu_1=mu_1max*mu_1;



//a_1=1;
//a_2=1;
//a_3=1;
//f_0=p1/p3;
//f_N=1;


N[i+1]=N[i]*(1+p1*a_1-p3*f_0*f_N*a_3);

D[i+1]=D[i]*exp(-mu_0-mu_1)+f_0*(p2*a_2+2*p3*a_3*f_N)*N[i];

Q[i+1]=Q[i] + Y_e*exp(delta*log(D[i]))+gamma*D[i]*(1-exp(-mu_0-mu_1))-m_r*(N[i]+D[i]);
Q[i+1]=Q[i+1]-m_d*N[i]*(p1*a_1 + f_0*(p2*a_2+p3*a_3*f_N));

if(Q[i+1]<0){
	Q[i+1]=0;
	}

Q_max=m_d*(N[i+1]+D[i+1]);
if(Q[i+1]>Q_max){
	Q[i+1]=Q_max;
	}
Size[i+1]=exp(-4.3839+.49713*log(N[i+1]+D[i+1]));
```

```
counter=counter+1;
if(counter==4000)
{
printf("%6d\t\%10.4f\t\%E\t\%E\t\%E\t\%1E\t\%E\t\%E\t\%E\t\%10.4f\t\%E\t\%10.4f\n",i,Size[i],q,Q_max,a_1*p1,a_2*f_0*p2,a_3*f_0*f_N*p3,mu_1,f_N,f_0,D[i],N[i]/(N[i]+D[i]));
counter=0;
}

fprintf(fptr,"%6d\t\%10.4f\t\%10.4f\t\%10.4f\t\%10.4f\t\%10.4f\t\%10.4f\t\%10.4f\%10.4f\t\%10.4f\n",i,Size[i],a_1*p1,a_2*f_0*p2,a_3*f_0*f_N*p3,mu_1,N[i]/(N[i]+D[i]),Y_e,f_0,f_N);
//end of i loop
}
fclose(fptr);

//************************************************************************************************************************************




//************************* finally generate some example activity multipliers**********
//printf("First generate some example activity multipliers and mu_1\n");
q_1=10;
q_2=40;
q_3=70;
q_4=90;
q_5=1.5*q_4;
q_mu=0.5*q_1;


q_12=0.5*(q_1+q_2);
q_23=0.5*(q_2+q_3);
q_34=0.5*(q_3+q_4);
q_45=0.5*(q_4+q_5);




fptr=fopen("Example_Activities.txt","w");
for(i_q=1;i_q<=150;i_q=i_q+1)
{
        //printf("%6d\n",i_q);

q=1.0*i_q;

//a_1=exp( (q-q_12)/sig_1);
//a_1=a_1/(1+a_1);

//a_2=exp( (q-q_23)/sig_2);
//a_2=a_2/(1+a_2);

//a_3=exp( (q-q_34)/sig_3);
//a_3=a_3/(1+a_3);

//mu_1=exp( (q-q_4)/sig_mu);
//mu_1=1/(1+mu_1);
```

```
if(q<q_1)
{
	a_1=0;
}
if(q>=q_1)
{
	qp=q-q_1;
a_1=qp*qp/(qp*qp + (q_12-q_1)*(q_12-q_1));
}

if(q<q_2)
{
	a_2=0;
}

if(q>=q_2)
{
	qp=q-q_2;
a_2=qp*qp/(qp*qp + (q_23-q_2)*(q_23-q_2));
}

if(q<q_3)
{
	a_3=0;
}
if(q>q_3)
{
	qp=q-q_3;

a_3=qp*qp/(qp*qp + (q_34-q_3)*(q_34-q_3));
}



if(q>10*q_1)
{
	mu_1=0;
}


if(q<q_mu)
{
	mu_1=1;
}
if(q>=q_mu)
{
if(q<=10*q_1)
{

	mu_1=(q_1-q_mu)*(q_1-q_mu)/(  (q_1-q_mu)*(q_1-q_mu)+ (q-q_mu)*(q-q_mu) );
}
}


//printf("%10.4f\t\%10.4f\t\%10.4f\t\%10.4f\t\%10.4f\n",q,a_1,a_2,a_3,mu_1);
fprintf(fptr,"%10.4f\t\%10.4f\t\%10.4f\t\%10.4f\t\%10.4f\n",q,a_1,a_2,a_3,mu_1);
}
fclose(fptr);
```

```
//now also show example of f_N and f_D
printf("f_D example\n");
i_y=15;
alpha[i_y]=(1/Dbar[i_y])*((p3/p1)-1);
N_c=f_N_mult*Nbar[i_y];
sig_N=f_N_mult*Nbar[i_y];

fptr=fopen("f_D.txt","w");
for(i_d=1;i_d<100;++i_d)
{
d=i_d*Dbar[i_y]/(100.);
f_D_ex=1/(1+alpha[i_y]*d);
//printf("%10.4f\t\%10.4f\n",100*d/(Dbar[i_y]),f_D_ex);
fprintf(fptr,"%10.4f\t\%10.4f\n",100*d/(Dbar[i_y]),f_D_ex);
}
fclose(fptr);


printf("f_N example\n");
fptr=fopen("f_N.txt","w");
for(i_n=1;i_n<=100;++i_n)
{
n=i_n*Nbar[i_y]/100.;
f_N_ex=exp((n-N_c)/sig_N);
f_N_ex=f_N_ex/(1+f_N_ex);
//printf("%10.4f\t\%10.4f\n",100*n/(Nbar[i_y]),f_N_ex);
fprintf(fptr,"%10.4f\t\%10.4f\n",100*n/(Nbar[i_y]),f_N_ex);
}
fclose(fptr);


        return 0;
}



//************************************************************************************************************************
//************************************************************************************************************************

//**************************************End of C Script for the Simplified Model****************************************

//************************************************************************************************************************
//************************************************************************************************************************





//************************************************************************************************************************
//************************************************************************************************************************

//**************************************R Script for the Simplified Model****************************************

//************************************************************************************************************************
//************************************************************************************************************************
```

```
A <-read.table("~marco/Example_Activities.txt", header=FALSE)
B <-read.table("~marco/NDQ4 Steady State.txt", header=FALSE)
C <-read.table("~marco/Grow.txt", header=FALSE)
D <-read.table("~marco/Excision.txt", header=FALSE)
E <-read.table("~marco/Xray.txt", header=FALSE)
F <-read.table("~marco/f_D.txt", header=FALSE)
G <-read.table("~marco/f_N.txt", header=FALSE)



# first --  feedback functions and sample activities
d=F[,1]
f_d=F[,2]

n=G[,1]
f_N=G[,2]

q=A[,1]
a_1=A[,2]
a_2=A[,3]
a_3=A[,4]
mu_1=A[,5]

#quartz()
#plot(n,f_N,type="l",lwd=3)
#quartz()
plot(d,f_d,type="l",lwd=3)


#quartz()
#par(mfrow=c(2,1))

#plot(q,a_1,type="l",lwd=3)
#lines(q,a_2,type="l",lwd=3)
#lines(q,a_3,type="l",lwd=3)

#plot(q,mu_1,type="l",xlim=c(0,20),ylim=c(0,0.005))

#plot(q,mu_1,type="l",lwd=3)




#second -- steady state size
quartz()
Y=B[,1]
Size_bar=B[,2]

plot(Y,Size_bar,type="l",xlab="Food level",ylab="Steady state size (mm)");


#third -- growth and regrowth dynamics


quartz()

t=C[,1]
Size=C[,2]
a_1=C[,3]
```

```
a_2=C[,4]
a_3=C[,5]
mu_1=C[,6]
frac_N=C[,7]
Y=C[,8]
f_D=C[,9]
f_N=C[,10]

#size
par(mfrow=c(2,1))
plot(t/10000,Y,type="l",xlab="Scaled time",ylab="Food (relative value)",lwd=3,ylim=c(240,380))
plot(t/10000,Size,type="l", xlab="Scaled time", ylab="Size (mm)",ylim=c(5,25),lwd=3)



quartz()

#activities
par(mfrow=c(4,1))

plot(t/10000,Y,type="l",ylab="Food (relative value)",lwd=3)
plot(t,a_1,type="l",ylim=c(0,0.00075),ylab="Activity")
plot(t,a_2,type="l",ylim=c(0,0.00075),ylab="Activity",col="red")
plot(t,a_3,type="l",ylim=c(0,0.00075),ylab="Activity",col="blue")
#lines(t,mu_1,type="l",col="green")


quartz()
#fraction N
par(mfrow=c(2,1))
plot(t/10000,Y,type="l",xlab="Scaled time", ylab="Food (relative value)")
plot(t/10000,frac_N,type="l",xlab="Scaled time", ylab="Fraction neoblasts",ylim=c(0,0.5),lwd=3)

quartz()
#last two feedback functions
par(mfrow=c(3,1))
plot(t/10000,Y,type="l",ylab="Food (relative value)",xlab="Scaled time",lwd=3)
plot(t/10000,f_D,type="l",ylab="Feedback from differentiated cells",xlab="Scaled time",lwd=3)
plot(t,f_N,type="l",ylab="Feedback from neoblasts",xlab="Scaled time",lwd=3)




#fourth -- excision experiment


t=D[,1]
Size_exc=D[,2]
a_1=D[,3]
a_2=D[,4]
a_3=D[,5]
mu_1=D[,6]
frac_N=D[,7]
f_D=D[,9]
f_N=D[,10]



quartz()
```

```
plot(t/10000,Size_exc,type="l",ylab="Size (mm)",ylim=c(10,24),xlim=c(6,10),lwd=3,xlab="Scaled time")


quartz()

#activities
par(mfrow=c(3,1))

plot(t,a_1,type="l",ylim=c(0,0.00075),ylab="Activity",xlim=c(60000,80000))
plot(t,a_2,type="l",ylim=c(0,0.00075),ylab="Activity",col="red")
plot(t,a_3,type="l",ylim=c(0,0.00075),ylab="Activity",col="blue")

quartz()
#fraction N and feedback functions
par(mfrow=c(3,1))
plot(t/10000,frac_N,type="l",ylim=c(0,0.5),lwd=3,xlab="Scaled time",ylab="Fraction neoblasts")


#last two feedback functions
plot(t/10000,f_D,type="l",ylab="Feedback from differentiated cells",xlab="Scaled time",lwd=3,ylim=c(0.85,0.95))
plot(t/10000,f_N,type="l",ylab="Feedback from neoblasts",xlab="Scaled time",lwd=3,ylim=c(0.73,0.74))


#fifth --xray experiment


t=E[,1]
Size_exc=E[,2]
a_1=E[,3]
a_2=E[,4]
a_3=E[,5]
mu_1=E[,6]
frac_N=E[,7]
f_D=E[,9]
f_N=E[,10]



quartz()
plot(t,Size_exc,type="l",ylab="Size (mm)",ylim=c(1,30),xlim=c(60000,100000),lwd=3)


quartz()

#activities
par(mfrow=c(3,1))

plot(t,a_1,type="l",ylim=c(0,0.00075),ylab="Activity",xlim=c(60000,80000),lwd=3)
plot(t,a_2,type="l",ylim=c(0,0.00075),ylab="Activity",col="red",lwd=3)
plot(t,a_3,type="l",ylim=c(0,0.00075),ylab="Activity",col="blue",lwd=3)

quartz()
#fraction N and feedback functions
par(mfrow=c(3,1))
plot(t/10000,frac_N,type="l",ylim=c(0.2,0.4),ylab="Fraction of neoblasts",lwd=3,xlab="Scaled time")
```

```
#last two feedback functions
plot(t/10000,f_D,type="l",ylab="Feedback from differentiated cells",xlab="Scaled time",ylim=c(0.88,0.94),lwd=3)
plot(t/10000,f_N,type="l",ylab="Feedback from neoblasts",xlab="Scaled time",ylim=c(0.62,0.74),lwd=3)



//*******************************************************************************************************************
//*******************************************************************************************************************

//************************************End of R Script for the Simplified Model**************************************

//*******************************************************************************************************************
//*******************************************************************************************************************
```
